# Small molecule allosteric modulation of the adenosine A_1_ receptor

**DOI:** 10.3389/fendo.2023.1184360

**Published:** 2023-06-26

**Authors:** Anh T. N. Nguyen, Quan L. Tran, Jo-Anne Baltos, Samantha M. McNeill, Diep T. N. Nguyen, Lauren T. May

**Affiliations:** ^1^ Drug Discovery Biology, Monash Institute of Pharmaceutical Sciences, Monash University, Parkville, VIC, Australia; ^2^ Department of Information Technology, Faculty of Engineering and Technology, Vietnam National University, Hanoi, Vietnam

**Keywords:** adenosine, A1 receptor, allosteric modulation, G protein-coupled receptor, structure-activity relationship, structure-function relationship

## Abstract

G protein-coupled receptors (GPCRs) represent the target for approximately a third of FDA-approved small molecule drugs. The adenosine A_1_ receptor (A_1_R), one of four adenosine GPCR subtypes, has important (patho)physiological roles in humans. A_1_R has well-established roles in the regulation of the cardiovascular and nervous systems, where it has been identified as a potential therapeutic target for a number of conditions, including cardiac ischemia-reperfusion injury, cognition, epilepsy, and neuropathic pain. A_1_R small molecule drugs, typically orthosteric ligands, have undergone clinical trials. To date, none have progressed into the clinic, predominantly due to dose-limiting unwanted effects. The development of A_1_R allosteric modulators that target a topographically distinct binding site represent a promising approach to overcome current limitations. Pharmacological parameters of allosteric ligands, including affinity, efficacy and cooperativity, can be optimized to regulate A_1_R activity with high subtype, spatial and temporal selectivity. This review aims to offer insights into the A_1_R as a potential therapeutic target and highlight recent advances in the structural understanding of A_1_R allosteric modulation.

## Introduction

The adenosine A_1_ receptor (A_1_R) belongs to the adenosine family of G protein-coupled receptors (GPCRs). The A_1_R is highly expressed in the central nervous system (CNS), particularly in the cerebral cortex, cerebellum, basal ganglia, thalamus, hypothalamus, midbrain, pons, medulla oblongata, hippocampal formation, spinal cord, white matter, and amygdala (1–4). In the periphery, A_1_R has moderate expression in the heart (5), parathyroid gland, salivary gland, pancreas, kidney, testis, placenta, and spleen (2, 6–9).

A_1_R regulate numerous physiological events. A_1_R activation reduces heart rate and contractility and confers potent cardioprotection following ischemia-reperfusion injury (10, 11). A_1_R inhibit presynaptic neurotransmitter release and induce neuronal hyperpolarisation at postsynaptic terminals (12). Additional physiological functions influenced by A_1_R activation include sleep regulation, inhibition of insulin release, and reducing renal blood flow (13–16). This review aims to offer insights into the importance of the A_1_R as a therapeutic target and will address recent advances in A_1_R allosteric ligands as a promising mechanism to selectively modulate A_1_R activity with spatial and temporal selectivity.

## Endogenous adenosine

Endogenous adenosine is a ubiquitous signaling molecule, acting *via* both autocrine and paracrine mechanisms to modulate physiology. Adenosine concentrations are largely maintained by dynamic but tightly regulated enzymatic processes that drive its formation, degradation, and transport. In the extracellular space, hydrolysis of adenine nucleotides can increase local adenosine concentrations. The enzyme CD39 (also known as ectonucleoside triphosphate diphosphohydrolase) converts extracellular ATP and ADP to form AMP, which can then be hydrolyzed by the enzyme CD73 (ecto-5′-nucleotidase) to form adenosine (17–19). Extracellular adenosine degradation involves the deamination of adenosine to inosine by adenosine deaminase (20). Intracellular adenosine can be produced through the conversion of AMP to adenosine by intracellular nucleotidases, primarily 5’-nucelotidases (21), or through the hydrolysis of S-adenosylhomocysteine (SAH) to adenosine and homocysteine by SAH-hydrolase (22). Intracellular adenosine degradation involves the conversion of adenosine to AMP by adenosine kinase (23). Throughout the body, the presence of bidirectional equilibrative nucleoside transporters 1 and 2 (ENT 1 & 2) preserve the concentration of adenosine both intra- and extracellularly *via* facilitated diffusion (24, 25). High adenosine concentrations can be maintained against a concentration gradient by unidirectional concentrative nucleoside transporters 1 and 2 (26).

Overall, these biochemical processes maintain the extracellular concentration of adenosine in a reported range of approximately 20-300 nM (27–29) However, in conditions associated with limited oxygen availability, including hypoxia, ischemia, cellular stress and damage, the local concentration of extracellular adenosine can dramatically rise to micromolar concentrations (30–33). As such, while concentrations of extracellular adenosine are tightly regulated under physiological conditions, coordinated mechanisms facilitate sensitive and dynamic responses under disease conditions.

## A_1_ receptor: a vital GPCR in health and disease

A_1_R typically modulates physiological effects through coupling to G_i/o_ proteins (34). Cardiac effects of A_1_R activation include a reduction in heart rate, through direct Gβγ-coupling to inwardly rectifying potassium channels or Gα_i/o_ inhibition of β-adrenoceptor-mediated cAMP production, antiarrhythmic effects, and atrial contractility (35, 36). Adenosine is used in the clinic to treat supraventricular tachycardia (37). Furthermore, A_1_R activation during ischemia-reperfusion injury promotes significant cardioprotection through stimulation of cardioprotective pathways, such as the reperfusion injury salvage kinase pathway (38–42). Cardiac overexpression of A_1_R in transgenic mice has been shown to confer significant protection against ischemia-reperfusion injury, an effect that was suggested to be mediated by mitochondrial $K_{ATP}^{+}$ channel activation (31, 43–45). The challenge for the development of cardioprotective agents that enhance A_1_R activity remains the need to separate the cardioprotective signaling from unwanted hemodynamic effects. Adenosine and the non-selective agonist AMP579 entered clinical trials to treat myocardial infarction (46–48). Unfortunately, the agonist doses administered in these trials were limited to avoid on-target adverse effects. Neladenoson, an A_1_R partial agonist, was evaluated in heart failure patients with reduced ejection fraction (HFrEF) or preserved ejection fraction (HFpEF) (49, 50). Neither clinical trial identified a significant drug effect on the primary endpoint, likely due to the partial agonism of neladenoson. However, recent advances in understanding GPCR biology, such as biased agonism and allostery, provide the opportunity to fine-tune receptor activity to overcome these limitations (11, 51).

Neuronal A_1_R activation can induce hyperpolarization and inhibition of neurotransmitter release, dampening neuronal excitability and promoting neuroprotection (3, 52). The A_1_R also plays an important role in cognition and can regulate sleep (16). Systemic administration of adenosine in animals reduces pain sensations through adenosine receptor stimulation, particularly A_1_R (53, 54). Enhanced A_1_R activation in the spinal cord of rats with neuropathic pain conferred significant analgesic effects (54, 55), likely through modulation of potassium channels (56) that reduced neuronal activity in sensory nerve terminals (57). An A_1_R positive allosteric modulator, T-62, was used in clinical trial to treat pain associated with postherpetic neuralgia (ClinicalTrials.gov Identifier: NCT00809679). Unfortunately, this trial was terminated due to a subset of patients having asymptomatic, transient elevations in liver transaminases. This trial highlighted the requirement for more efficacious and tailored modulation of A_1_R activity, alongside a detailed mechanistic understanding of the drug-receptor-effector interactions, to facilitate clinical translation.

Additional peripheral effects of the A_1_R include decreased lipolysis and increased glucose uptake in adipocytes, reduced renal blood flow and tubuloglomerular feedback, inhibition of renin release, increased sodium and water reabsorption, and inhibition of insulin and glucagon release (58–66). Given the role of the A_1_R in the renal system, it has been suggested that the A_1_R may represent a therapeutic target, as its stimulation can protect against acute renal ischemia-reperfusion injury (67), whilst A_1_R antagonism may be useful in treating acute renal disorders in patients with congestive heart failure (68–70). Clinical trials assessed whether A_1_R inhibition can improve renal function in heart failure patients and airway hyperreactivity and late allergic response in asthmatics (71, 72). The A_1_R antagonist rolofylline was evaluated in a large multicenter, double-blind, placebo-controlled Phase III clinical trial in acute heart failure patients to prevent the commonly associated deterioration of renal function. The trial was conducted following positive results from smaller trials that indicated A_1_R antagonism increased the glomerular filtration rate and urine output (73). However, the Phase III trial observed no benefit of rolofylline with respect to the primary clinical composite endpoint or renal function (74). Moreover, rolofylline was associated with an increased incidence of seizure, an effect attributed to the A_1_R antagonism.

A_1_R activation has been suggested to promote inflammation, bronchoconstriction, and mucous secretion (75). Thus, inhibition of the A_1_R may be useful in patients with asthma (76). Promising results have been presented for Phase IIa trials in mild-to-moderate atopic asthmatics, where the ability of the orally available A_1_R antagonist PBF-680 to inhibit the late allergic response or adenosine monophosphate airway hyperresponsiveness was assessed (71, 72). Recruitment is ongoing for another Phase IIa trial of PBF-680 in patients with chronic obstructive pulmonary disease (ClinicalTrials.gov Identifier: NCT05262218). As such, the A_1_R remains a promising novel therapeutic target for several respiratory diseases.

Clearly, A_1_R represents a promising therapeutic target for the treatment of major global health burdens. However, the transition of A_1_R small molecule agonists and antagonists into the clinic has thus far failed. Common challenges of A_1_R drug discovery include the design of highly subtype-selective molecules due to conservation in the adenosine-binding pocket across the adenosine receptor family and modulation of A_1_R signaling beyond the scope of the desired effect (10, 11, 54, 77, 78). Unwanted outcomes of A_1_R activation can include bradycardia, atrioventricular block, a reduction in atrial contractility and sedation, whereas over-inhibition of A_1_R can increase seizure liability (79, 80). Such on-target unwanted effects are typically associated with targeting the A_1_R orthosteric binding site. In contrast, drugs targeting the allosteric binding site can be tailored for an optimal therapeutic profile, providing exquisite selectivity of effect, acting to “fine-tune” endogenous agonist tone in a tissue- and disease-specific manner.

## Allosteric modulation to “tailor” A_1_R activity with tissue- & disease-specificity

A_1_R possess allosteric sites that are spatially distinct from the adenosine-binding pocket, known as the orthosteric site (81, 82). Allosteric ligands can influence the binding and/or function of orthosteric ligands. Positive allosteric modulators (PAMs) enhance, whereas negative allosteric modulators (NAMs) inhibit, the affinity and/or efficacy of an endogenous ligand for its cognate receptor. Neutral allosteric ligands (NAL) have neutral cooperativity with the orthosteric ligand. Allosteric ligands have the capacity to stabilize the active or inactive receptor conformation thereby having intrinsic efficacy in the absence of orthosteric ligand, adding an additional level of texture to their profile (83).

**Figure 1:**
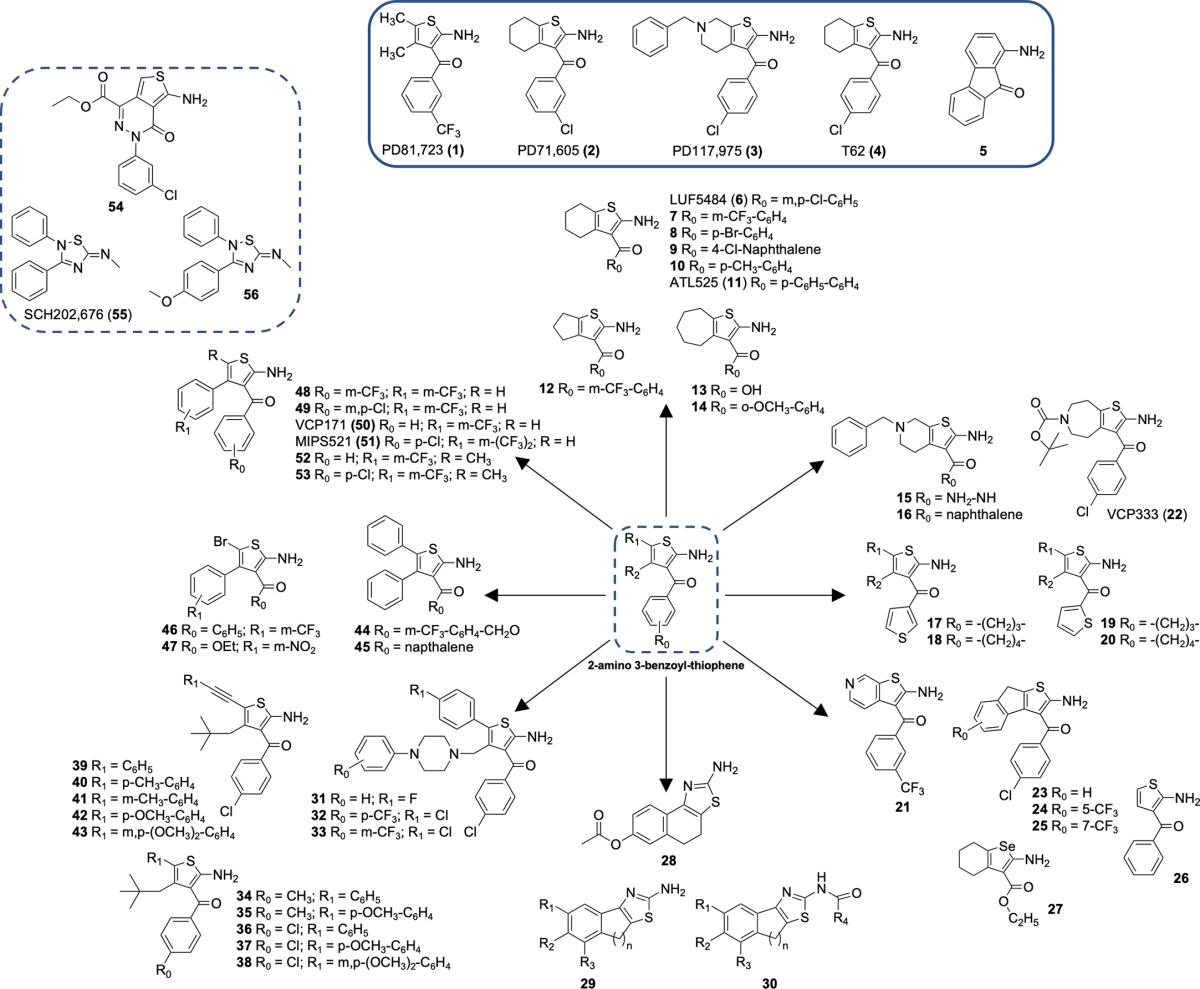
A select subset of adenosine A1 receptor allosteric modulators.

The A_1_R was the first GPCR for which a PAM was identified. This seminal discovery identified a series of 2-amino-3-benzoylthiophenes, including the well characterized A_1_R PAM, PD 81,723 (1; 2-amino-4,5-dimethyl-3-thienyl-[3-(trifluoromethyl)phenyl]methanone) (**Figure 1**) (84, 84). Pharmacological studies demonstrated that several 2-amino-3-benzoylthiophenes had an allosteric mechanism of action at the A_1_R, decreasing the rate of agonist dissociation and enhancing agonist binding and function. Subsequent studies have demonstrated that A_1_R PAMs enhance the actions of endogenous adenosine in the heart, including cardioprotection (85–87) and atrioventricular nodal function (88, 89). A_1_R PAMs have also been shown to promote anti-nociception, selectively reducing hypersensitivity (54, 55, 90, 91). Recent breakthroughs in the structural understanding of A_1_R PAMs provide the opportunity to optimize PAM design to enhance allosteric enhancer potency whilst removing the adverse effects observed for T-62 (54). As such, the rational design of allosteric modulators will facilitate the future therapeutic translation of this class of small molecules.

### Advantages of allosteric modulation

A_1_R allosteric binding sites provide a mechanism to target the A_1_R with subtype, spatial, and temporal selectivity. As such, A_1_R allostery is a promising approach to overcome current therapeutic limitations of orthosteric ligands. Allosteric ligands can display high subtype selectivity as a direct consequence of their allosteric nature. Allosteric sites are spatially distinct from the orthosteric site, which typically exhibit significant sequence conservation across receptor subtypes due to evolutionary pressure (81, 92, 93). Allosteric sites typically show greater sequence divergence compared to orthosteric sites, facilitating the design of small molecules with high subtype-selectivity and, as such, minimizing the potential for off-target side effects.

The reciprocal nature of the binding cooperativity enables allosteric modulators to “sense” the (patho)physiological concentration of the endogenous agonist within a specific tissue (81, 94). A_1_R allosteric modulators will predominantly exert their effects at the time and location of significant cytoprotective adenosine release and have little effect in the presence of a low adenosine concentration (95). This feature is particularly promising for the development A_1_R PAMs that modulate receptor activity with tissue and disease-specificity, as the local adenosine concentration has been shown to increase significantly for several disease conditions associated with cellular stress or hypoxia (96, 97). Under these conditions, PAMs will primarily act at the site of injury, avoiding the classical on-target systemic side effects associated with prototypical A_1_R agonists.

The influence of an allosteric modulator on orthosteric ligand binding is saturable, a feature that contrasts competitive interactions. The influence of an allosteric modulator on orthosteric ligand affinity is defined by the cooperativity between the allosteric and orthosteric sites when both ligands are co-bound to the receptor. This property enables allosteric modulators to ‘fine-tune’ orthosteric ligand activity by scaling up or down the binding cooperativity as appropriate whilst avoiding the over-stimulation or complete inhibition often observed with orthosteric agonists and antagonists, respectively (81).

Advantages of allosteric ligands that deserve greater exploration at the A_1_R include the ability of allosteric ligands to promote biased signaling and regulate receptor trafficking from the allosteric site. Allosteric ligands may stabilize unique receptor conformations, influencing the spectrum of pathways stimulated or inhibited by an orthosteric ligand. Indeed, A_1_R PAMs have been shown to stimulate biased agonism in the absence of an orthosteric ligand or mediate biased allosteric modulation of an orthosteric agonist (98). Although relatively unexplored, A_1_R PAMs have been suggested to cause less receptor desensitization compared to A_1_R orthosteric agonists, an effect that may be therapeutically beneficial (99). Such features of A_1_R allosteric ligands require further in-depth exploration due to potential therapeutic implications.

## Structure-activity relationship of A_1_R Allosteric Modulators

### 2-amino 3-benzoylthiophenes as A_1_R PAMs

A_1_R is the first GPCR for which the allosteric modulators were identified in the 1990s (84) The first representatives were three compounds PD81723 (**1**), PD71605 (**2**), and PD117975 (**3**). Since then, benzoylthiophene derivatives have been intensively investigated (**Table 1**, **Figure 1**) for their allosteric mechanism of action, typically slowing the rate of orthosteric agonist dissociation and enhancing agonist activity in functional assays (84). A_1_R PAM 2-amino-3-benzoylthiophene (2A3BT) scaffolds, represented by PD81723 (**1**), typically display significant A_1_R selectivity and the best ratio of enhancement to inhibition (100, 120). The extension of this family, followed by additional analogs represented by T62 (**4**), had substituents in 4- and 5-positions in the thiophene moiety bridged by a methylene chain (100, 120, 121). However, these compounds were inhibitors at higher concentrations and displayed significant intrinsic activity by causing a functional response in the absence of an agonist.

**Table 1:** An overview of structural modifications of A_1_R allosteric modulators.

Compound	Main Scaffold	Modification of 2-amino-3-benzoyl-thiophene							Reference
		*2- position*	*3- position*	*4- position*	*5- position*	*Bridging 4- and 5-*	*Benzoyl*	*Non-thiophene*	
**1** (PD81,723)	thiophene								(84)
**2** (PD71,605)	tetrahydrobenzo[*b*]thiophene					x	x		
**3** (PD117,975)	4,5,6,7-tetrahydrothieno[2,3-*c*]pyridine					x	x		
**4** (T62)*	4,5,6,7-tetrahydrobenzo[*b*]thiophene					x	x		(100)
**5**	9*H*-fluoren-9-one							x	(84)
**6** (LUF5484)*	4,5,6,7-tetrahydrobenzo[*b*]thiophene					x	x		(100)
7*	4,5,6,7-tetrahydrobenzo[*b*]thiophene					x	x		(100)
8*	4,5,6,7-tetrahydrobenzo[*b*]thiophene					x	x		(101)
**9**	3-naphthoylthiophenes					x	x		(101)
**10**	4,5,6,7,8,9-hexahydrocycloocta[*b*]thiophenes					x	x		(102)
**11** (ATL525)	2-amino-3-benzoyl-thiophenes					x	x	x	(103)
**12**	4,5,6,7,8,9-hexahydrocycloocta[*b*]thiophenes					x	x		(102)
**13**	5,6,7,8-tetrahydrocyclohepta[b]thiophene		x			x			(104)
**14**	5,6,7,8-tetrahydro-4*H*-cyclohepta[*b*]thiophene					x	x		(105)
**15**	4,5,6,7-tetrahydrothieno[2,3-*c*]pyridines		x	x	x	x			(106)
**16**	4,5,6,7-tetrahydrothieno[2,3-*c*]pyridine					x	x		(107)
**17**	5,6-dihydro-4*H*-cyclopenta[*b*]thiophene					x	x		(108)
**18**	4,5,6,7-tetrahydrobenzo[b]thiophene					x	x		
**19**	5,6-dihydro-4*H*-cyclopenta[*b*]thiophene					x	x		
**20**	4,5,6,7-tetrahydrobenzo[*b*]thiophen					x	x		
**21**	thieno[2,3-*c*]pyridine					x	x		(109)
**22** (VCP333)	4,5,7,8-tetrahydro-6*H*-thieno[2,3-*d*]azepine					x	x		(109)
**23**	thiophene					x	x		(110)
**24**	thiophene					x	x		
**25**	thiophene					x	x		
26*	thiophene			x	x		x		(101)
27*	selenophene		x			x		x	(111)
28*	thiazolium		x			x		x	(112)
**29**	1,2,4-thiadiazole	x	x	x	x		x	x	(113)
**30**	1,2,4-thiadiazole	x	x	x	x		x	x	(113)
31*	thiophene			x	x		x		(114)
32*	thiophene			x	x		x		
33*	thiophene			x	x		x		
34*	thiophene			x	x		x		(114)
35*	thiophene			x	x		x		
36*	thiophene			x	x		x		
37*	thiophene			x	x		x		
38*	thiophene			x	x		x		
39*	thiophene			x	x		x		(114)
40*	thiophene			x	x		x		
41*	thiophene			x	x		x		
42*	thiophene			x	x		x		
43*	thiophene			x	x		x		
44*	4,5-diphenylthiophene			x	x		x		(115)
45*	4,5-diphenylthiophene			x	x		x		
46*	4-phenylthiophenes			x	x		x		
47*	4-phenylthiophenes		x	x	x				
**48**	thiophene			x	x		x		(116)
**49**	thiophene			x	x		x		
**50**	thiophene			x	x		x		
**51**	thiophene			x	x		x		
**52**	thiophene			x	x		x		
**53**	thiophene			x	x		x		
**54**	3,4-dihydrothieno[3,4-**d**]pyridazine		x	x					(117)
**55** (SCH-202676)	1,2,4-thiadiazole	x	x	x	x		x	x	(118)
**56**	1,2,4-thiadiazole	x	x	x	x		x	x	(119)

* more potent than PD81723 as allosteric enhancers of agonist binding and/or function,

"x" denotes the substitutent has been modified.

SAR studies on the 2A3BT scaffold have identified general structural requirements for the PAM effects (122). The omission of the 2-amino and 3-keto groups of the 2-aminothiophene core resulted in a notable decrease of allosteric enhancer (AE) activity, highlighting the importance of these groups for PAM activity. An intramolecular hydrogen bond between these two groups is proposed to create an additional coplanar ring with the thiophene ring in the active conformation of the PAMs. Supporting this hypothesis, 1-aminofluoren-9-one (**5**), which conformationally locked the amino and keto groups with a hydrogen bond, was reported to have good AE activity (122). 4- and 5- substituents in 2A3BT, such as large hydrophobic alkyl to aryl groups, have been shown to increase the AE activity of 2A3BT derivatives (116), suggesting the inclusion of such substituents in the design of more potent A_1_R PAMs.

### SAR of the thiophene ring of A_1_R PAMs

The thiophene ring in the PAM scaffold with substituents in the 4- and 5-position plays an important role in conferring allosteric activity. Increased A_1_R AE activity was observed for compounds with a long carbon chain bridging the 4- and 5-positions, such as LUF 5484 (**6**), **7-20**, or **21-25** (98, 100, 102, 104, 105, 107–110). The optimal size of the 4,5-cycloalkyl ring varied from five to seven-membered rings to maintain PAM activity measured by *in vitro* dissociation kinetic binding assays (105). Compound **26**, without 4- and 5-substituents in the thiophene ring, displayed lower allosteric agonism than PD81723 (**1**) and increased antagonism (101).

Selenophenes and thiazoles have been characterized as A_1_R PAMs. Preliminary data for 2-aminoselenophene-3-carboxylate (**27**) suggested superior A_1_R AE activity compared to PD81723 (**1**), although the compound was unstable under mildly acidic conditions (111). Introduction of a nitrogen atom into the thiophene ring was also shown to retain A_1_R PAM activity, as demonstrated by the synthesis of a series of 2-aminothiazolium salts, which identified **28** as a relatively potent and efficacious A_1_R PAM (112). However, synthesis of another series of 2-aminothiazoles (**29, 30**) did not display A_1_R PAM activity (113). In fact, several 2-aminothiazoles inhibited A_1_R activity.

The presence of hydrophobic groups at the 4-position of thiophene ring, such as arylpiperazine moieties (**31-33**) or neopentyl (**34-43**), with **39-43** having an addition (hetero)aryl moiety in the 5-position of the thiophene ring, are suggested to be important for hydrophobic interactions within the A_1_R allosteric binding site (123–125). Such compounds had superior AE potency compared to the well-studied PD81723 (**1**). Furthermore, the two-carbon linker between hetero aryl or akyl moiety at the 5-position of thiophene ring (**39-43**) allowed for exploration of the hydrophobic domains within the A_1_R allosteric pocket (125).

### SAR of 3-benzoyl moiety of A_1_R PAMs

Numerous modifications in the 3-benzoyl ring of the original PD81723 (**1**) scaffold have been investigated, some of which are well tolerated. For example, substitutions of the 3-benzoyl moiety as trifluoromethyl in PD81723 (**1**); chloro in LUF5484 (**6**), or phenyl in ATL525 (**11**) have been suggested to improve the magnitude of positive allosteric modulation. 3-benzoyl moiety modification combined with the introduction of large hydrophobic domains in the 4-position of the thiophene ring enhanced compound AE activity, suggesting the A_1_R allosteric pocket can accommodate a range of substituents.

Substitution of the 3-benzoyl group by a 3-carboxylate (**13**) or 3-hydrazide (**15**) can maintain A_1_R PAM activity comparable to PD81723 (**1**), although these compounds were reported as a novel class of A_1_R antagonists that recognized the allosteric site at higher concentrations (105, 107). Replacement of the phenyl ring at the 3-position with a naphthoyl (**9, 16, 30**) (107, 115) or unsubstituted thienyl ring (**17-20**) (108) was well tolerated with a number of derivatives favoring A_1_R PAM activity. 2-aminothiophene with a carboxylic acid (**13**) or ester (**27, 47**) substituents in the 3-position or incorporation of phenyl groups in the 4- and 5-position retained A_1_R AE activity in functional assays (104, 111, 115). Only continuous replacement of 3-benzoyl moiety with a benzyl-ester (**44**) or 1-naphthoyl group (**45**); or the addition of a halo substituent (Br) in the 5-position of the thiophene (**46, 47**) significantly improved the A_1_R enhancer effect (115).

In addition, the nature of the 3-benzoyl moiety has been shown to influence A_1_R PAM bias signaling (116). Among the evaluated series of potent A_1_R PAMs (**48-53**), MIPS521 (**51**) displayed high AE activity alongside considerable allosteric agonism (116). Compared to VCP171 (**50**), which induced weak analgesic effects in rats (55), MIPS521 (**51**) stimulated significant analgesia efficacy in an *in-vivo* rat model, in the absence of notable side effects (54). Structural derivatives of **51**, specifically **52** and **53**, displayed biased profiles (116). The structure of **52** and **53** differed only by the presence of one electron-withdrawing group (*p*-chloro) on the 3-benzoyl ring. However, they displayed distinct A_1_R signaling profiles in functional assays, with **52** behaving as a biased allosteric agonist and **53** conferring biased allosteric modulation (116)

Interestingly, a PD81723 (**1**) derivative with bridged 3- and 4- positions displayed A_1_R antagonism at low concentrations, but PAM activity at higher concentrations (**54**) (117). Similarly, the scaffold 2-amino-4,5,6,7-tetrahydrothieno[2,3-*c*]pyridines scaffold with a carbohydrazide substitution at the 3- position (**15**) was reported as a novel class of A_1_R antagonists that recognized the allosteric site at relatively high concentrations (106).

### Thiadiazole compounds

A thiadiazole compound, SCH-202676 (**55**) (N-(2,3-diphenyl-[1,2,4]-thiadiazol-5-(2*H*)-ylidene)-methanamine) was reported to act as a non-selective allosteric inhibitor of several Family A GPCRs, including A_1_R (118). Synthesis of 2,3,5-substituted [1,2,4]-thiadiazole analogues of SCH-202676 identified (**56**), which appeared to act as an allosteric inhibitor of agonist binding (119). However, subsequent studies suggested that these compounds acted as non-selective protein modifiers, with their GPCR effects resulting from sulfhydryl modification. Some amiloride analogs have also been reported to act as A_1_R negative allosteric modulators (NAMs), increasing the dissociation rate of the antagonist [^3^H]DPCPX without affecting the dissociation rate of [^3^H]R-PIA (126, 127).

## A_1_R allosteric binding site

### Mutagenesis and computational modeling studies

Prior to the determination of a high-resolution structure of A_1_R bound to an allosteric modulator, efforts have been made to map the location of the A_1_R allosteric site using indirect approaches such as mutagenesis and computational modeling. Several structure-function studies have identified important residues for A_1_R allosteric modulation, with the second extracellular loop (ECL2) playing a crucial role (128–130). Alanine substitution of residue E172^ECL2^ caused a significant decrease in the binding affinity for the unoccupied A_1_R of two allosteric modulators, PD81723 and VCP171 (130). This study also predicted that residues involved in allosteric ligand intrinsic efficacy were relatively conserved between the two modulators and the hydrogen-bonding networks within A_1_R extracellular vestibule may facilitate the transmission of cooperativity between orthosteric and allosteric sites.

### A_1_R X-ray crystallography and cryo-electron microscopy structures

Inactive and active state A_1_R structures have been solved, providing important insights into A_1_R ligand binding and activation. A_1_R structures in complex with the orthosteric antagonists DU172 and PSB36 (PDB: 5UEN and 5N2S, respectively) (77, 131) or the endogenous agonist, adenosine (PDB: 6D9H) (78) revealed common interaction with conserved residues F171^ECL2^ (via a π- π stacking interaction) and N254^6.55^ (via a double hydrogen bond) superscript denoting Ballesteros-Weinstein residue numbering (132). In both inactive and active structures, A_1_R ECL2 was found to adopt a unique conformation compared to its relative A_2A_R with a longer helix and almost perpendicular to the plane of the membrane. The inactive A_1_R is characterized by a wider extracellular vestibule (compared to the A_2A_R structures) that may hold an orthosteric and allosteric site. Residue T270^7.35^ within this pocket was found to be responsible for the selectivity of the antagonist DU272 (77). Due to the movement of the transmembrane domain 1 and 2, the orthosteric binding site of the active A_1_R was found to have a contraction on the extracellular surface compared to the inactive A_1_R (78). An all-atom Gaussian accelerated molecular dynamics (GaMD) simulation was performed using A_1_R inactive structure (PDB: 5UEN) to predict the binding modes of two A_1_R PAMs, PD71723 **1** and VCP171 **27** (133). The simulation further supported the role of residue E172^ECL2^ as a key binding determinant of the two PAMs and showed that the presence of PAMs stabilized the bound agonist within the transmembrane bundle.

Most recently, the cryoEM structure of A_1_R-G_i2_ complex co-bound with adenosine and the PAM (MIPS521; **51**) was solved (PDB: 7LD3) (54). MIPS521 bound to a unique, extrahelical lipid-facing pocket, harbored by hydrogen bonds between its 2-amino substituent of the thiophene ring and residues in TM6 and TM7 (S246^6.47^ and L276^7.41^). The binding site was well supported by site-directed mutagenesis studies where mutation of these two residues to alanine significantly decreased MIPS521 binding affinity, owing to the loss of a hydrogen bond with the 2-amino group (54). All-atom GaMD simulations in combination with deep learning and free energy profiling workflow (GLOW) of the active A_1_R structure co-bound to adenosine in the presence or absence of MIPS521 were employed to determine conformational changes mediated by A_1_R activation and allosteric modulation (134). Interestingly, the study confirmed that ECL2 has a significant impact on A_1_R allosteric modulation.

## Future applications of computational advances in the development of A_1_R allosteric ligands as novel therapeutics

### Artificial intelligence to facilitate the development of novel selective and drug like A_1_R PAMs

Artificial intelligence (AI), including machine learning and deep learning methods, are widely used in GPCR drug discovery. In 2021, 34% of publications related to GPCR drug discovery mentioned AI, and the number increased to more than 40% in 2022 based on Google Scholar search results. AI applications vary from simply detecting properties and outcomes (e.g., active/inactive states) to recognizing the characteristics and patterns of activity (e.g., finding functional sites) and generation of *de novo* drug candidates. Predicting properties is often done *via* classification models using traditional machine learning methods or deep learning techniques like convolutional neural network (CNN) and recurrent neural network (RNN) (134, 135). Generative models, either trained *via* deep learning or reinforcement learning, are the most advanced in the field (136–141). They can be used not only to generate *de novo* structures (i.e., design new drug candidates) but also to predict the properties and inspect the functional sites of a structure. In these generative methods, the chemical space of small molecules is constructed and explored iteratively under constrained objectives, namely desired 3D structures or desired functions.

### Multistage virtual screening

Compared with the traditional experimental high-throughput screening, virtual screening (structure-based or ligand-based approaches) has emerged as a more direct and rational drug discovery approach to screen large libraries of chemical structures. Approximately 60 GPCR structures with small-molecule allosteric ligands have been determined using X-ray crystallography or cryo-EM (142, 143). GPCR allosteric sites have been identified in a range of locations, including within the seven transmembrane domains (corticotropin-releasing factor receptor 1, metabotropic glutamate receptors 1,2, and 5, smoothened receptor, luteinizing hormone-choriogonadotropin receptor, calcium-sensing receptor), within the extracellular vestibule (M2 and M4 muscarinic acetylcholine receptor, protease-activated receptor-2), outside the seven-transmembrane domain (C5a anaphylatoxin chemotactic receptor 1, GPR40 receptor, P2Y1 receptor, glucagon receptor, protease-activated receptor-2, glucagon-like peptide-1 receptor, adenosine A_1_ receptor, cannabinoid CB1 receptor), overlapping with cholesterol-binding sites (bile acid receptor, D1 dopamine receptor, glucagon-like peptide 1 receptor), or on intracellular surface (β_2_ adrenergic receptor, CC chemokine receptor 2 and 9) (142, 143). Such structural determination has provided key insights into allosteric binding across various GPCRs, which in turns aids the discovery of new allosteric modulators using the structure-based drug discovery (SBDD) approach. Several SBDD studies have successfully employed to find new allosteric modulators for GPCRs, including M2 muscarinic acetylcholine receptors (144, 145), glucagon-like peptide 1 receptor (146–148), metabotropic glutamate receptor 5 (149), and proton-sensing receptors GPR68 and GPR65 (150). These studies demonstrate the power of SBDD to identify new allosteric modulators for GPCRs, paving the way for the development of novel therapeutics with improved selectivity and reduced side effects.

The recent breakthroughs in solving A_1_R structures have enriched our understanding of A_1_R ligand binding and signal transduction and offered a great opportunity to leverage SBDD approaches. The A_1_R structure co-bound with a PAM and agonist has provided significant structural detail, which will facilitate the design of novel A_1_R allosteric modulators as new therapeutic agents. Molecular dynamics (MD) simulations and site-directed mutagenesis can enhance our understanding of the activation mechanism and generate multiple conformations of dynamic receptors co-bound with orthosteric and allosteric ligands. The rapid development of pharmacophore and molecular docking methods also enabled the screening of very large databases from millions to billions of molecules *via* ligand-based drug discovery (LBDD) approaches. Combining SBDD and LBDD, the multistage virtual screening approaches are now possible for drug discovery (135).

Multistage methods can be designed as a workflow of several filters against large databases. Workflows have been developed to use deep learning to classify A_1_/A_2A_R antagonists among ChemDiv database, then pharmacophore modeling and docking for additional filtering, and finally MD to observe drug-receptor interactions to support compound activity (135). Alternative workflows can be designed using MD to gain insights into drug candidate interactions at A_1_R, then applying pharmacophore modeling to predict key ligand features and screening of small molecule databases using molecular docking. A different workflow could first integrate a generative deep learning method to obtain ligands or drug candidates by exploring chemical space, then apply a permutation of virtual screening methods and property prediction methods to further filter through the candidate sets. The profound understanding of A_1_R structures, SAR of A_1_R PAMs, alongside the growing accessibility of data sources, public AI tools, and source codes, have paved the way for the imminent discovery of novel A_1_R PAMs.

## Author contributions

AN and LM designed the structure of the manuscript. AN, QT prepared **Figure 1** and **Table 1**. All authors contributed to writing and reviewing the article and approved the submitted version.
